# Evaluating the Quality of Patient Handoff Communication in the Department of Medicine at Dongola Specialized Hospital

**DOI:** 10.7759/cureus.95955

**Published:** 2025-11-02

**Authors:** Abdalftah Noureldin Abdalftah Osman, Basil Hamed, Mohammed Ali Mohammed Ali, Aboaagla Abdalbagi Ali Awad Alkareem, Fatima Essamaldin Altahir Mohamed Alsharif, Ammar Abdelrahman Hussein Dousa, Abdelmuniem Siddig Mohamed Ahmed, Malaz Siddeg Younis, Elkhatab Abdulrahman Mustafa Abdulrahman, Mohammed Osman Ahmed Osman, Mohammed Siddeg Younis, Ibrahim Yousif Ibrahim Elhagelbashir, Samah Elhussein Mohamed Ahmed, Mohamed Abdalla Elawad Wedatalla, Faheema Essamaldeen Altaher Mohammed Alsharif, Maali Yousif Mustafa Idris, Ezzat Khiery, Mohamed Elsheikh Farah Mohamed Elhassan, Abdulrahman Abbas Yusuf Mohammed, Samah Omer Abdallah Ibrahim

**Affiliations:** 1 General Practice, Dongola Specialized Hospital, Dongola, SDN; 2 Paediatrics, National Ribat University, Dongola, SDN; 3 Internal Medicine, Dongola Specialized Hospital, Dongola, SDN; 4 General Practice, Response Plus Medical Services, Abu Dhabi, ARE; 5 Internal Medicine, Port Sudan Teaching Hospital, Port Sudan, SDN; 6 Medicine, University of Gezira, Wad Madani, SDN; 7 Paediatrics and Child Health, Dongola Specialized Hospital, Dongola, SDN

**Keywords:** communication, handover, i-pass, patient safety, quality improvement, sudan

## Abstract

Background

Communication failure at the point of handoff is one of the most significant preventable medical errors, especially in resource-scarce settings. Worldwide, the use of the I-PASS (Illness severity, Patient summary, Action list, Situation awareness and contingency planning, and Synthesis by receiver) tool has been shown to improve the accuracy and consistency of handovers of patients. The quality improvement audit was to assess and optimize patient handoff through the structured utilization of the I-PASS tool for Dongola Specialized Hospital in Sudan.

Methods

A prospective three-cycle audit, the Department of Medicine of Dongola Specialized Hospital was subjected to three rounds of quality improvement audit between 2 November 2023 and 11 September 2025. All clinical handoffs by medical officers, registrars, and house officers were observed. Standards for the audit were derived from the I-PASS system, covering illness severity, patient summary, action list, situational awareness, and synthesis by receiver. Compliance was recorded through structured observation checklists, and focused intervention included role-plays, training workshops, and standard checklists. Chi-square testing was used for statistical analysis.

Results

Cycle 1 (n = 37) demonstrated poor baseline compliance: illness severity (21.6%), summary statements (10.8%), and continuous assessment (8.1%). Meaningful improvements were observed at Cycle 2 (n = 74), such as illness severity (68.9%) and summary statements (93.2%), although timeline/ownership (41.9%) and receiver synthesis (2.7%) declined. In Cycle 3 (n = 86), continued and comprehensive improvements were achieved: illness severity (96.5%), summary statements (100%), continuous assessment (98.8%), and plan documentation (95.3%). Receiver synthesis improved to 88.4%, question-asking to 80.2%, and ownership to 100%. Chi-square analysis confirmed statistically significant improvements in all parameters (p < 0.0001).

Conclusion

Orderly implementation of the I-PASS communication framework significantly increased the safety, thoroughness, and precision of Dongola Specialized Hospital's patient transitions. Incremental improvements noted over three rounds reiterated the value of educational activities, follow-up audits, and institutional support for institutionalizing structured communication methodologies. Extension of the I-PASS model to additional departments and medical facilities in Sudan can potentially improve patient safety in a precarious healthcare environment.

## Introduction

Patient handoff is a vital component of clinical practice, ensuring the safe transfer of information and responsibility between healthcare providers. Failures in handoff communication are among the leading causes of preventable medical errors and adverse patient outcomes worldwide [1]. To address this issue, standardized tools such as I-PASS (Illness severity, Patient summary, Action list, Situation awareness and contingency planning, and Synthesis by receiver) and SBAR (Situation, Background, Assessment, Recommendation) were developed to improve clarity and consistency of communication across various clinical settings [2,3].

The I-PASS handoff tool has proven to be one of the most evidence-based methods for reducing communication failures. Its use in multicenter studies demonstrated significant decreases in medical errors and improvements in handoff accuracy and reliability [4,5]. The tool’s adaptability has been confirmed in specialties such as pediatrics and surgery, where it enhanced the effectiveness and safety of patient transitions [3,6].

Sudan’s healthcare system has faced unprecedented strain due to ongoing conflict and resource shortages, contributing to communication breakdowns and compromised patient safety [7]. Previous Sudanese quality-improvement projects, such as audits on discharge summaries and radiology request forms, have shown that structured documentation can markedly improve compliance and quality of care [8-11]. These findings highlight the urgent need for structured handoff systems like I-PASS in local hospitals.

This audit aimed to evaluate the implementation and impact of the I-PASS framework on handoff quality over three audit cycles in the Department of Medicine at Dongola Specialized Hospital. The project represents one of the first applications of I-PASS in a Sudanese specialized hospital and seeks to provide evidence for its feasibility and effectiveness in low-resource settings.

## Materials and methods

The quality improvement audit was performed at Dongola Specialized Hospital, Sudan, and it was aimed at assessing and enhancing patient handoff communication based on the I-PASS handover framework in the Department of Medicine. The cross-sectional design that was used was prospective and spanned three audit cycles between 2 November 2023 and 11 September 2025, with the aim of evaluating compliance with structured handoff practices and quantifying the effects of targeted interventions. All house officers, medical officers, and registrars who worked in the department within the time periods of the audit were the study population.

Audit standards and criteria

Audit standards were defined based on what the I-PASS handoff program was, which is a standardized communication tool to minimize medical errors and improve patient safety. The parameters that were assessed were illness severity; patient summary (summary statement, events leading to admission, hospital course, ongoing assessment, and plan); action list (tasks, timeline, ownership); situation awareness and contingency planning (awareness of patient condition and anticipatory guidance regarding complications); and synthesis by receiver (read-back/teach-back, clarifying questions, restating key action items) [12,13].

Data collection

All the handoffs were monitored and recorded with the help of a structured questionnaire that aimed at capturing compliance with the essential elements of communication. The use of the tool was trained and was performed in real time by the observers in clinical handoffs. Observers were aware of the ongoing audit but blinded to the specific audit cycle outcomes and statistical goals to minimize observer bias. Each observer followed identical procedures using standardized checklists across all cycles to ensure consistency.

Questionnaire

The questionnaire, as designed, was based on the I-PASS handoff framework and the SBAR model of communication. It included 15 questions in five domains: Illness severity (one item), Patient summary (five items), Action list (three items), Situational awareness and contingency planning (three items), and Synthesis by receiver (three items). All items were evaluated with a dichotomous scale (yes/no) with the help of direct observation at handoffs. To establish content validity, the panel of patient safety medical educators and clinicians was able to review it. To remove ambiguity and impracticability, the tool was tested on a small sample of handoffs. The internal consistency was high (Cronbach = 0.82), and inter-rater reliability was high (Cohen = 0.78). The questionnaire is presented in its entirety in the Appendices.

Cycle 1: Baseline Audit and Problem Identification (2 November 2023-15 March 2024)

Cycle 1 set down the standards for compliance with the I-PASS. The observations during handover were carried out, and the compliance in each of the domains was documented using a structured questionnaire. Findings showed that there was a great deficiency in the areas of low compliance with illness-severity reporting, low use of summary statements, low contingency planning, and virtually no read-back or teach-back. The main challenges were also found to be a shortage of formal training and a lack of uniform handover procedures.

Intervention: Training and Education (16 March 2024-19 March 2024)

Due to root-cause analysis, specific interventions were introduced in order to meet the gaps identified. Workshops were provided to the doctors, emphasizing I-PASS elements and the role of organized communication. Role-play exercises and practical demonstrations were made to achieve the correct use of the framework in patient handovers. The wards also had standardized checklists and visible reminders that would promote real-time adherence.

Cycle 2: Audit and Outcome Assessment of Intervention (20 March 2024-30 December 2024)

Cycle 2 evaluated the same areas with the use of the same tools. Several areas were significantly improved, especially in the application of summary statements, contingency planning, and recording of the critical actions. Adherence to illness-severity reporting improved significantly, and the knowledge of continuous measures improved. But receiver synthesis, and particularly read-back practice, and task ownership were still subject to consistent underperformance.

Cycle 3: Long-Term Improvement and Observation (15 January 2025-11 September 2025)

The sustainability of these improvements was tested in Cycle 3. The findings indicated sustained improvement in the majority of I-PASS areas, and significant improvements in illness-severity reporting, continuing assessment, contingency planning, and receiver synthesis. The progress at the three cycles was an indicator of increased compliance by the staff with the organized communication practices that underscored the efficacy of the continuous monitoring and re-audit as measures of ensuring high standards of patient care.

Statistical analysis

Data were analyzed using IBM SPSS Statistics (version 26.0, IBM Corp., Armonk, NY). Descriptive statistics summarized compliance rates across I-PASS domains. Comparative analysis using chi-square tests assessed differences in compliance between audit cycles (Cycle 1 vs. Cycle 2, Cycle 2 vs. Cycle 3, and overall Cycle 1 vs. Cycle 3). Statistically significant improvement was observed across all parameters (p < 0.0001). A p-value of <0.05 was considered statistically significant. Table 1 summarizes the detailed compliance rates and statistical outcomes.

**Table 1:** Audit Results of I-PASS Handoff Parameters (Cycle 1-3)

Parameter	Cycle 1 (n = 37)	Cycle 2 (n = 74)	Cycle 3 (n = 86)	Improvement (1→2)	Improvement (2→3)	Improvement (1→3)	Chi-square	p-value	Note of Improvement	Improvement Category
Illness Severity	8/37 (21.6%)	51/74 (68.9%)	83/86 (96.5%)	47.3%	27.6%	74.9%	72.69	0.0000	Improved by 74.9%	Significant improvement (74.9%)
Summary Statement	4/37 (10.8%)	69/74 (93.2%)	86/86 (100.0%)	82.4%	6.8%	89.2%	144.14	0.0000	Improved by 89.2%	Significant improvement (89.2%)
Event Leading Up to Admission	5/37 (13.5%)	70/74 (94.6%)	86/86 (100.0%)	81.1%	5.4%	86.5%	142.71	0.0000	Improved by 86.5%	Significant improvement (86.5%)
Hospital Course	0/37 (0.0%)	19/74 (25.7%)	65/86 (75.6%)	25.7%	49.9%	75.6%	74.37	0.0000	Improved by 75.6%	Significant improvement (75.6%)
Ongoing Assessment	3/37 (8.1%)	41/74 (55.4%)	85/86 (98.8%)	47.3%	43.4%	90.7%	99.54	0.0000	Improved by 90.7%	Highest improvement (90.7%)
Plan	6/37 (16.2%)	62/74 (83.8%)	82/86 (95.3%)	67.6%	11.6%	79.1%	92.99	0.0000	Improved by 79.1%	Significant improvement (79.1%)
To-Do List	37/37 (100.0%)	74/74 (100.0%)	86/86 (100.0%)	0.0%	0.0%	0.0%	N/A	N/A	Improved by 0.0%	No improvement (0.0%)
Timeline and Ownership	29/37 (78.4%)	31/74 (41.9%)	86/86 (100.0%)	-36.5%	58.1%	21.6%	70.43	0.0000	Improved by 21.6%	Moderate improvement (21.6%)
Know What’s Going On	9/37 (24.3%)	44/74 (59.5%)	83/86 (96.5%)	35.1%	37.1%	72.2%	68.15	0.0000	Improved by 72.2%	Significant improvement (72.2%)
Plan for What Might Happen	6/37 (16.2%)	26/74 (35.1%)	84/86 (97.7%)	18.9%	62.5%	81.5%	98.51	0.0000	Improved by 81.5%	Significant improvement (81.5%)
Receiver Summarizing What Was Heard	14/37 (37.8%)	2/74 (2.7%)	76/86 (88.4%)	-35.1%	85.7%	50.5%	118.72	0.0000	Improved by 50.5%	Significant improvement (50.5%)
Ask Questions	0/37 (0.0%)	2/74 (2.7%)	69/86 (80.2%)	2.7%	77.5%	80.2%	129.39	0.0000	Improved by 80.2%	Significant improvement (80.2%)
Key Actions/To-Do Items	17/37 (45.9%)	1/74 (1.4%)	70/86 (81.4%)	-44.6%	80.0%	35.4%	103.14	0.0000	Improved by 35.4%	Moderate improvement (35.4%)

Ethical considerations

Patient confidentiality was ensured by anonymizing all the data. The audit was officially enrolled as a quality-improvement project within the leadership of the hospital and was therefore not subject to the need for separate informed consent. The hospital has the Institutional Review Board (IRB) that provided ethical approval.

## Results

The implementation of the I-PASS structured handoff protocol at Dongola Specialized Hospital was evaluated across three audit cycles (October 2024-June 2025). Statistically significant improvements were observed in all measured domains, confirming progressive enhancement in communication quality and patient safety.

Cycle 1: baseline

Baseline documentation was poor (n = 37). Illness severity was recorded in 21.6% of handoffs, summary statements in 10.8%, and events leading to admission in 13.5%. Hospital course was undocumented, ongoing assessment noted in 8.1%, and plan documentation in 16.2%. Although “to-do” lists were consistently present (100.0%), ownership (78.4%) and receiver summarization (37.8%) were suboptimal, indicating a lack of structured communication practices.

Cycle 2: post-intervention

Following targeted workshops and checklist implementation (n = 74), notable improvements were achieved in most areas: illness severity (68.9%), summary statements (93.2%), and events leading to admission (94.6%). However, despite overall gains, receiver synthesis declined to 2.7%, and timeline/ownership dropped to 41.9%, suggesting difficulty in sustaining new communication behaviors immediately after training. Question-asking began to appear (2.7%) during this phase, marking early engagement in two-way communication.

Cycle 3: sustained improvement

In the re-audit phase (n = 86), nearly all domains achieved high or full compliance. Illness severity (96.5%), summary statements (100.0%), and ongoing assessment (98.8%) showed substantial and sustained improvement. Receiver synthesis demonstrated remarkable recovery to 88.4% after reinforcement training, while question-asking increased to 80.2%, indicating improved interaction between handoff participants. Ownership and timeline documentation reached 100.0%, and plan documentation improved to 95.3%, confirming integration of the I-PASS framework into routine practice.

Summary

All I-PASS elements showed progressive improvement across cycles, with the greatest gains in ongoing assessment (↑ 90.7%) and summary statements (↑ 89.2%). Receiver synthesis initially declined post-intervention but improved substantially in Cycle 3, reflecting the impact of reinforcement and feedback sessions.

**Figure 1:**
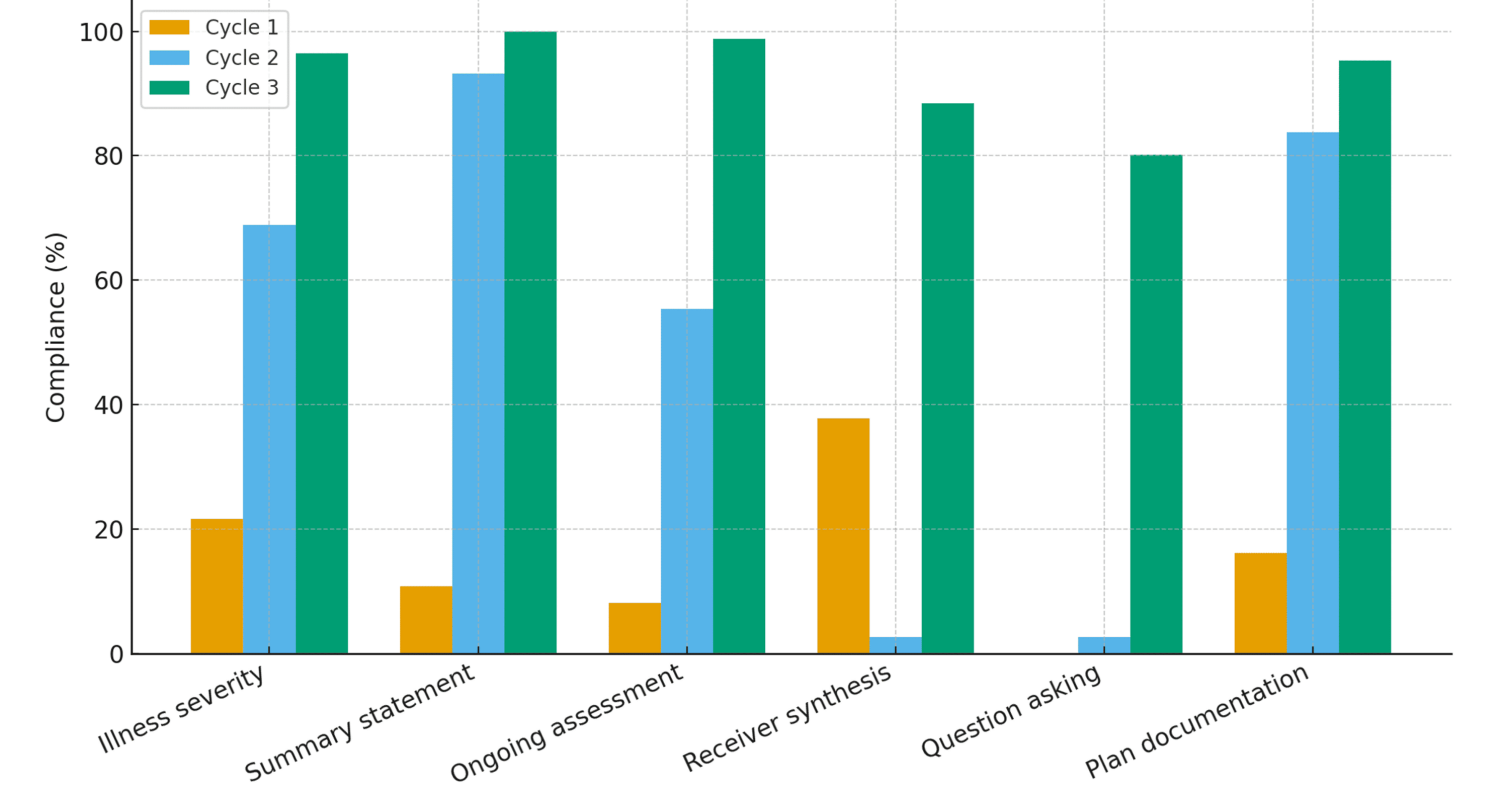
Percentage Compliance Across Audit Cycles Using the I-PASS Framework at Dongola Specialized Hospital

Figure 1 visually illustrates the progressive percentage improvements across the three cycles, highlighting key domains such as illness severity, summary statements, and receiver synthesis.

## Discussion

The quality improvement audit that was performed identified that the rigorous use of the I-PASS handoff tool significantly improved the accuracy, completeness, and reliability of patient transfers at Dongola Specialized Hospital. Across three iterations of audit, adherence showed improvement in almost all aspects of the I-PASS model, with many of the metrics reaching adherence rates above 90% by Cycle 3. These outcomes validate the effectiveness of I-PASS in enhancing communication and patient safety, even in the challenging environment of resource-constrained, high-demand Sudan.

Major improvements were identified in summary statements, reporting of illness severity, and ongoing assessment, progressing from poor initial documentation to virtual full compliance by Cycle 3. These results are in agreement with multicenter studies showing that I-PASS decreases avoidable medical errors and improves situational awareness for medical professionals [1]. A trial described in the New England Journal of Medicine identified a marked reduction in medical errors after implementation of I-PASS [2], and subsequent accounts have confirmed its versatility for use in many specialties and institutions [3,4].

Receiver synthesis (teach-back/read-back) and question-asking enhancements took longer to develop. Both elements declined during early Cycle 2, yet grew significantly by Cycle 3, with over 80% compliance. These outcomes reflect the behavioral and cultural challenges of integrating active two-way communication habits into clinical practice, especially in low-resource and hierarchical healthcare systems where junior staff may hesitate to clarify or repeat senior instructions. Overcoming these barriers required repeated reinforcement, role modeling, and leadership engagement factors that were crucial for sustained behavioral change. Comparable outcomes were observed in oncology centers, where provider task forces improved adherence [5], and in surgical and pediatric units where reinforcement methods enhanced receiver engagement [4,6].

Ownership and timeline areas also demonstrated transitional challenges. Compliance dipped during Cycle 2 and subsequently reached 100% during Cycle 3, mirroring trends seen in comparable quality improvement projects in Sudan and internationally, where structured tools initially disrupt workflows before producing sustainable improvement [3,7]. Results of this audit are in agreement with prior Sudanese projects, such as implementation of SBAR tools in pediatric care [3], audits of radiology request slips [8], discharge summary audits [9,10,14], and standardizing acute fracture documentation [11]. These projects collectively demonstrate that structured communication facilitates compliance with standards, minimizes variability, and strengthens patient safety. Unlike those studies, the present audit uniquely focused on real-time bedside handoffs, the step most prone to misunderstanding and of greatest clinical significance to patient outcomes. The benefit of structured communication is even more vital in fragile healthcare environments like Sudan, where ongoing conflict and limited resources magnify the risk of communication breakdowns [7].

This study has some limitations. It was performed in a single department, limiting generalizability. Although observers were pre-trained and used standardized tools, observer awareness of audit phases could have influenced performance (Hawthorne effect). Furthermore, while compliance markedly improved, the audit did not assess objective outcome measures such as reduction in adverse events, readmissions, or hospital stay duration. Future research should integrate these clinical outcome metrics to demonstrate the direct impact of I-PASS on patient safety and healthcare quality.

Finally, this audit demonstrates that I-PASS is practical and adaptable, with strong potential to improve the quality of hospital handoffs in Sudan. Repeated training, regular reassessment, and institutional support are essential for successful adoption. Expanding the program to additional departments and hospitals could further strengthen patient safety and foster a culture of structured, accountable communication across the healthcare system [9-14].

## Conclusions

The introduction of the I-PASS handoff tool at Dongola Specialized Hospital led to marked improvements in the quality, consistency, and safety of patient handovers over three audit cycles. By the third cycle, nearly all I-PASS domains achieved high compliance, reflecting the positive impact of structured education, checklists, and continuous re-audit.

While the findings highlight I-PASS as a practical and sustainable model in a resource-limited setting, the study’s scope was restricted to a single department. Broader multi-department and multi-center validation is needed to confirm generalizability and to evaluate direct effects on patient outcomes. Continued institutional commitment to training and monitoring will be essential for sustaining gains and expanding the culture of safe communication across Sudan’s healthcare system.

## Appendices

**Table 2:** Structured I-PASS Handoff Questionnaire

Domain	Item No.	Question	Response (Yes/No)
Illness Severity	1	Was the patient’s illness severity stated (stable/unstable/watcher)?	☐ Yes ☐ No
Patient Summary	2	Was a one-line summary statement provided?	☐ Yes ☐ No
	3	Were events leading to admission described?	☐ Yes ☐ No
	4	Was the hospital course summarized?	☐ Yes ☐ No
	5	Was an ongoing assessment provided?	☐ Yes ☐ No
	6	Was the plan of care documented?	☐ Yes ☐ No
Action List	7	Were tasks to be completed specified?	☐ Yes ☐ No
	8	Was a timeline for tasks provided?	☐ Yes ☐ No
	9	Was task ownership assigned?	☐ Yes ☐ No
Situation Awareness and Contingency Planning	10	Was the current patient condition discussed?	☐ Yes ☐ No
	11	Were anticipated problems or complications identified?	☐ Yes ☐ No
	12	Were contingency plans or triggers for escalation outlined?	☐ Yes ☐ No
Synthesis by Receiver	13	Did the receiver perform read-back/teach-back?	☐ Yes ☐ No
	14	Did the receiver ask clarifying questions?	☐ Yes ☐ No
	15	Were key action items restated?	☐ Yes ☐ No

## References

[1] Starmer AJ, Spector ND, O'Toole JK (2023). Implementation of the I-PASS handoff program in diverse clinical environments: A multicenter prospective effectiveness implementation study. J Hosp Med.

[2] Franco Vega MC, Ait Aiss M, George M (2024). Enhancing implementation of the I-PASS handoff tool using a provider handoff task force at a comprehensive cancer center. Jt Comm J Qual Patient Saf.

[3] Adam MH, Ali HA, Koko A (2022). The use of the Situation, Background, Assessment, and Recommendation (SBAR) form as a tool for handoff communication in the pediatrics department in a Sudanese teaching hospital. Cureus.

[4] Wolinska JM, Lapidus-Krol E, Fallon EM, Kolivoshka Y, Fecteau A (2022). I-PASS enhances effectiveness and accuracy of hand-off for pediatric general surgery patients. J Pediatr Surg.

[5] Elamin A, Abdullah S, ElAbbadi A, Abdellah A, Hakim A, Wagiallah N, Ansah JP (2024). Sudan: from a forgotten war to an abandoned healthcare system. BMJ Glob Health.

[6] Starmer AJ, Spector ND, Srivastava R (2014). Changes in medical errors after implementation of a handoff program. N Engl J Med.

[7] Blazin LJ, Sitthi-Amorn J, Hoffman JM, Burlison JD (2020). Improving patient handoffs and transitions through adaptation and implementation of I-PASS across multiple handoff settings. Pediatr Qual Saf.

[8] Muhammed A, Mohammed Ahmed AB, Ahmed Mohmed MH (2025). Improving radiology request form compliance: a clinical audit at Prince Othman Digna Teaching Hospital, Sudan. Cureus.

[9] Abdalrahman IB, Mohammed MEH (2018). Improving paper-based discharge process; a continuous full-cycle quality improvement project in low resource setting. F1000Research.

[10] Hammad EA, Wright DJ, Walton C, Nunney I, Bhattacharya D (2014). Adherence to UK national guidance for discharge information: an audit in primary care. Br J Clin Pharmacol.

[11] Mohamed A, Muhammed A (2024). A study at Wad Madani, Sudan: are we documenting acute ankle fractures effectively?. Cureus.

[12] (2025). Tool: SBAR. https://www.ahrq.gov/teamstepps-program/curriculum/communication/tools/sbar.html.

[13] (2025). Tool: I-PASS. https://www.ahrq.gov/teamstepps-program/curriculum/communication/tools/ipass.html.

[14] Eissa AY, Mohamed Elhassan AZ, Ahmed AZ (2023). The quality of discharge summaries at Al-Shaab Hospital, Sudan, in 2022: the first cycle of a clinical audit. Cureus.

